# Sonodynamic therapy inhibits palmitate-induced beta cell dysfunction via PINK1/Parkin-dependent mitophagy

**DOI:** 10.1038/s41419-019-1695-x

**Published:** 2019-06-11

**Authors:** Tian Guo, Tianyang Liu, Yun Sun, Xianna Liu, Rongguo Xiong, He Li, Zhitao Li, Zhiguo Zhang, Zhen Tian, Ye Tian

**Affiliations:** 1 Department of Pathophysiology, Harbin Medical University, Harbin, 150081 China; 2 Laboratory of Photo- and Sono-theranostic Technologies and Condensed Matter Science and Technology Institute, Harbin Institute of Technology, Harbin, 150001 China; 3 Key Laboratory of Acoustic Photoelectric Magnetic Diagnosis and Treatment of Cardiovascular Diseases in Heilongjiang Province, Harbin, 150081 China; 4 Department of Cardiology, The First Affiliated Hospital, Cardiovascular Institute, Harbin Medical University, Harbin, 150001 China

**Keywords:** Mitophagy, Type 2 diabetes

## Abstract

In type 2 diabetes mellitus (T2DM), the overload of glucose and lipids can promote oxidative stress and inflammatory responses and contribute to the failure of beta cells. However, therapies that can modulate the function of beta cells and thus prevent their failure have not been well explored. In this study, beta cell injury model was established with palmitic acid (PA) to simulate the lipotoxicity (high-fat diet) found in T2DM. Sonodynamic therapy (SDT), a novel physicochemical treatment, was applied to treat injured beta cells. We found that SDT had specific effects on mitochondria and induced transient large amount of mitochondrial reactive oxygen species (ROS) production in beta cells. SDT also improved the morphology and function of abnormal mitochondria, inhibited inflammatory response and reduced beta cell dysfunction. The improvement of mitochondria was mediated by PINK1/Parkin-dependent mitophagy. Additionally, SDT rescued the transcription of PINK1 mRNA which was blocked by PA treatment, thus providing abundant PINK1 for mitophagy. Moreover, SDT also increased insulin secretion from beta cells. The protective effects of SDT were abrogated when mitophagy was inhibited by cyclosporin A (CsA). In summary, SDT potently inhibits lipotoxicity-induced beta cell failure via PINK1/Parkin-dependent mitophagy, providing theoretical guidance for T2DM treatment in aspects of islet protection.

## Introduction

With excess nutrition or too little exercise, an overabundance of glucose and free fatty acids (FFAs) accumulates in the body. Then, lipotoxicity induced by excess FFAs, an important pathogenesis of type 2 diabetes mellitus (T2DM), promotes oxidative stress and inflammation in beta cells^1–4^. As beta cell damage occurs, insulin secretion is gradually reduced, and hyperglycemia, in turn, stimulates oxidative stress, which increases damage to beta cells. The dysregulation of insulin secretion and the failure of beta cells eventually lead to T2DM and serious complications^5,6^. Thus, protecting the secretory function of beta cells is an important modality for the treatment of T2DM.

The treatment strategy for the control of blood glucose is mainly focused on two aspects, i.e., oral medication and insulin injection. Oral medications, such as sulfonylureas, can promote endogenous insulin secretion via different signaling pathways but are prone to cause severe hypoglycemia^7–9^. Exogenous insulin injection, especially short-term intensive insulin therapy for new-onset diabetes, allows beta cells to rest and regain function^10,11^. Although these interventions can partially control blood glucose or temporarily improve insulin secretion, the incidence of cardiovascular events caused by T2DM has not decreased significantly^12,13^. The intrinsic cause might be the lack of a modality to directly protect beta cells, and current modalities cannot inhibit the functional decline of beta cells^14,15^.

Autophagy is involved in the regulation of the physiological function of islet beta cells in T2DM^16^. Autophagy can remove misfolded proteins or damaged mitochondria induced by endoplasmic reticulum (ER) stress and plays a role reducing oxidative stress^17^. Defective autophagy is the underlying cause of diabetes, metabolic heart disease and lipid disorders^18–20^. For example, autophagy induced by rapamycin can recognize the toxic effect of lipids to protect the function of beta cells^21^. However, excessive autophagy damages beta cells as well as reduces their insulin secretion^22^. Therefore, modalities based on the protective effect of autophagy in beta cells for the control of T2DM still needs to be explored.

Sonodynamic therapy (SDT) exerts its biological effect by ultrasonically activating the sonosensitizer accumulated in target tissues. 5-Aminolevulinic acid (ALA) can serve as a sonosensitizer in multiple cell types, e.g., macrophages and tumor cells^23^. As a nontoxic, noninvasive treatment, SDT is widely applied in several diseases, such as tumors and atherosclerosis^23–25^; substantial therapeutic effects have been observed in many cancers, such as breast cancer, lung cancer and glioma^26,27^, as well as in atherosclerosis. For example, SDT could induce the apoptosis or autophagy in macrophages of atherosclerotic plaques, reverse necroptosis to apoptosis, reduce inflammation, stabilize plaques and inhibit the progression of atherosclerosis^28,29^. Currently, SDT has not been explored in the treatment of diabetes; thus, investigating whether SDT could protect beta cells treated with palmitic acid (PA), the most common saturated FFA, is theoretically valuable.

The current research applied SDT, a novel nontoxic and noninvasive modality, to treat beta cells with FFA-induced damage and to explore the protective role of SDT and autophagy in beta cells. This study of SDT provides experimental support for the treatment of T2DM.

## Materials and methods

### Cell line and reagents

The rat insulinoma beta cell line RIN-m5f was obtained from the China Center for Type Culture Collection (China). ALA (A7793), PA (P0500), PK11195 (C0424), N-acetylcysteine (NAC, A9165), Cyclosporin A (CsA, 239835), glucose (G7021), HEPES (H3375) and dimethyl sulfoxide (DMSO, D4540) were purchased from Sigma-Aldrich (USA); sodium bicarbonate (S118660), and ethanol were from Aladdin Bio-Chem Technology (China); sodium pyruvate (P0582) and glutamine (G0063) were from TCI Shanghai (China), and phosphate-buffered saline (PBS) was from Beyotime Biotechnology (China). ALA and NAC were dissolved in PBS, PA and CsA were dissolved in ethanol, and PK11195 was dissolved in DMSO as stock solutions. The final concentrations of ethanol or DMSO as vehicles in the culture medium were less than 0.05% and 0.1%, respectively.

### Cell culture and PA treatments

RIN-m5f beta cells were cultured in RPMI 1640 culture medium (Biological Industries, Israel) containing 11.1 mmol/l glucose, 10% (v/v) fetal bovine serum (FBS, Vitrocell, Uruguay), 100 IU/ml penicillin, 100 μg/ml streptomycin, 10 mmol/l HEPES, 1.5 g/l sodium bicarbonate, 1 mmol/l sodium pyruvate and 2 mmol/l glutamine at 37 °C under 5% CO_2_ and 95% humidified air. The culture medium was changed every 2 days until the cells grew to 80% confluence. Then, the cells were plated in 96-well plates (1 × 10^4^ cells/well), 24-well plates (2 × 10^5^ cells/well), or 35 mm diameter culture dishes (1 × 10^6^ cells/dish).

Beta cell injury model was established by coculturing beta cells with different concentrations of PA for 48 h, as previously described^30^. The optimal concentration of PA to induce beta cell injury was determined by cell viability assays. If necessary, SDT was performed at 24 h of PA treatment.

### Sonodynamic treatment

Except for the control group and ultrasound (US) group, beta cells were incubated with 1 mmol/l ALA for 9 h before ultrasound treatment. Then, the cells in the US and SDT groups were irradiated with a 1 MHz ultrasonic transducer (Harbin Institute of Technology, China) for 5–15 min. The ultrasonic transducer was placed horizontally while the bottom of the culture dish or plate in close contact with the center of the transducer through coupling agents. The range of ultrasound intensity was 0.1–0.5 W/cm^2^, the duty ratio was 10% and pulse repetition frequency of 100 Hz (i.e., pulse duration of 1 ms). The maximal pressure amplitude (0-peak pressure) (*p*_MAX_) can reflect the bioeffects of ultrasound to cells but independent of experimental environment, such as materials and dimensions of various well dish products^31^. *p*_MAX_ on the axis of culture dish center at different intensity was measured using a needle hydrophone (1 mm in diameter) (Onda, USA). The hydrophone was placed in culture medium at 0.75 mm (half wavelength) distance from the cells at the bottom of culture dish. The detected signal was registered on a digital oscilloscope (Tektronix, USA). The results are shown in Table S1. The exposure of cells to light was avoided during the whole procedure. Cells were harvested or detected 0.5–24 h after ultrasound treatment.

### Cell viability assay

Cell viability was measured with a Cell Counting Kit-8 (CCK-8, Dojindo, Japan) according to the manufacturer’s instructions. Briefly, CCK-8 solution was added to the wells (10 μl/well) and incubated at 37 °C for 2 h. Then, the OD was measured at a wavelength of 450 nm with a microplate reader (BioTek, USA). Data are normalized to 0 mmol/l ALA or 0 W/cm^2^ SDT.

### ALA-PpIX fluorescent detection

Beta cells were incubated with 1 mmol/l ALA for 0–24 h or 0–2 mmol/l ALA for 9 h. Then, the fluorescence of ALA-protoporphyrin IX (PpIX) was detected with a fluorescence spectrophotometer (Varian, USA) at λ_Ex/Em_ = 405/635 nm. Next, PicoGreen (Thermo Fisher, USA) staining was employed to quantitate double-stranded DNA (cell population). Cells were incubated with PicoGreen at 37 °C for 1 h following incubation with ddH_2_O for 1 h. The fluorescence intensity of PicoGreen was measured at λ_Ex/Em_ = 480/545 nm. The ALA-PpIX fluorescence was normalized to the cell population and corresponding control.

### ROS measurement

Intracellular and mitochondrial reactive oxygen species (ROS) levels were measured by DCFH-DA (Beyotime, China) and MitoSOX (Thermo Fisher, USA) staining, respectively. Briefly, cells were loaded with 10 μmol/l DCFH-DA (λ_Ex/Em_ = 488/525 nm) or 5 mmol/l MitoSOX (λ_Ex/Em_ = 510/580 nm) in serum-free medium for 10–20 min at 37 °C. Then, mitochondrial ROS production was measured with a fluorescence spectrophotometer (Varian, USA) in real time. Intracellular and mitochondrial ROS were also analyzed by a NovoCyte flow cytometer and NovoExpress software (ACEA, China) or imaged with a CCD camera connected to a fluorescence microscope (Olympus, Japan) (×200) at 24 h post SDT.

### Mitochondrial singlet oxygen measurement

Mitochondrial singlet oxygen (^1^O_2_) levels were measured by Si-DMA (Dojindo, Japan) staining. Si-DMA, a far-red fluorescence probe, can selectively detect ^1^O_2_. Briefly, cells were loaded with 100 nmol/l Si-DMA (λ_Ex/Em_ = 600/680 nm) in serum-free medium for 1 h at 37 °C. Next, cells were treated with ultrasound if necessary. Hoechst 33342 (2 μg/ml, Sigma-Aldrich, USA) was added to the medium to label cell nuclei at 37 °C for 10 min. Then, ^1^O_2_ production observed with a laser scanning confocal microscope (LSCM, Zeiss, Germany) (×400) at 0.5 h post SDT. Cells were protected from light throughout the process, except using LSCM to observe.

### Cell damage assay

Cell apoptosis was analyzed and interleukin-1β (IL-1β) secretion was measured to assess cell damage.

Cell apoptosis was measured with an Annexin V/PI Apoptosis Detection Kit (Dojindo, Japan). Cells were digested with trypsin and centrifuged at 600 × *g* and 4 °C for 5 min. After the cells were resuspended, each tube, containing 1 × 10^5^ cells stained with 5 μl of Annexin V-FITC and 5 μl of PI solution, was incubated for 15 min at room temperature. Data were collected with a flow cytometer. Annexin V-FITC was detected at λ_Ex/Em_ = 494/518 nm, and PI was detected at λ_Ex/Em_ = 535/617 nm.

The secretion of IL-1β was measured using a Rat IL-1β ELISA Kit (Neobioscience, China). Expression levels of apoptosis-related proteins and inflammatory factors were also explored by western blotting.

### Mitochondrial damage assay

Mitochondrial membrane potential (Δψm) was assessed with a JC-1 Kit (Beyotime Biotechnology, China) at 24 h post SDT. Cells were loaded with JC-1 staining solution at 37 °C for 20 min. Images of JC-1 fluorescence were acquired with a fluorescence microscope (Olympus, Japan) (×200). At low Δψm, JC-1 is a green-fluorescent monomer (λ_Ex/Em_ = 475/535 nm). At higher Δψm, i.e., normal Δψm, JC-1 forms red-fluorescent aggregates (λ_Ex/Em_ = 475/595 nm). Data are shown as a ratio of red-fluorescent cell number to green-fluorescent cell number.

The ultrastructure of mitochondria was observed with transmission electron microscopy (TEM, Hitachi, Japan) at 24 h post SDT. Cells were centrifuged at 2000 × *g* and 4 °C for 5 min to prepare cell pellets. Cell pellets were fixed with 2.5% glutaraldehyde and postfixed with 1% osmium tetroxide. Ultrathin sections were subsequently stained with uranyl acetate and examined using TEM (×15000).

### Detection of autophagy

Autophagosomes were labeled with a Cell Meter Autophagy Assay Kit (AAT Bioquest, USA) according to the manufacturer’s instructions. Briefly, autophagosomes were stained with Autophagy Blue solution at 0.5 h post SDT, and mitochondria were labeled with Mito-Tracker Green (MTG, Beyotime, China) at 37 °C for 30 min. Then, Hoechst 33342 (2 μg/ml, Sigma-Aldrich, USA) was added to the medium to label cell nuclei at 37 °C for 10 min. Autophagy Blue (λ_Ex/Em_ = 333/518 nm), MTG (λ_Ex/Em_ = 490/516 nm) and Hoechst 33342 (λ_Ex/Em_ = 355/465 nm) staining was observed with a fluorescence microscope (×400). Expression levels of autophagy-related proteins (e.g., LC3, PINK1 and Parkin) were measured by western blotting, and cell ultrastructure was observed with TEM (×15,000) at 0.5 h post SDT.

### Western blotting

Mitochondrial proteins were extracted with a Cell Mitochondria Isolation Kit (Beyotime, China) according to the manufacturer’s instructions. Immunoblotting of cell lysates and mitochondrial extracts was performed as previously described^28^. Primary antibodies against the following proteins were used: β-actin (1:2000, 66009–1-Ig, Proteintech, China), caspase-3 (1:1000, 19677–1-AP, Proteintech, China), B-cell lymphoma-2 (Bcl-2, 1:1000, ab59348, Abcam, USA), Bcl-2 associated X protein (Bax, 1:1000, ab182733, Abcam, USA), caspase-1 (1:1000, HPA003056, Sigma, USA), IL-1β (1:800, 12703, Cell Signaling Technology, USA), Cytochrome c oxidase IV (COXIV, 1:1000, 11242–1-AP, Proteintech, China), Microtubule-associated protein 1 light chain 3B (LC3B, 1:1000, L7543, Sigma, USA), PTEN-induced kinase 1 (PINK1, 1:1000, ab23707, Abcam, USA), and Parkin (1:1000, 14060–1-AP, Proteintech, China). HRP-linked antibodies (anti-rabbit IgG, 7074; anti-mouse IgG, 7076) were from Cell Signaling Technology (1:5000, USA). The blots were developed with ECL reagent (Merck, Germany), and densitometric analysis was performed using ImageJ software (NIH, USA).

### Real-time quantitative PCR

Cells were collected at 0.5 h post SDT and total mRNA was extracted from those cells using a Magnetic Bead-based RNA Isolation Kit (Bimake, USA) according to the manufacturer’s protocols. cDNA was synthesized by a PrimeScript RT reagent Kit (Takara, Japan). qPCR was performed using a Light Cycler 96 system (Roche, USA), using SYBR Premix Ex Taq II Kit (2×, Takara, Japan) and 500nmol/l specific primers and 10 ng cDNA in each reaction. The thermal recycling conditions used were as follows: initial denaturation step at 95 °C for 30 s, followed by 60 cycles of denaturation at 95 °C for 5 s, primer annealing and extension at 60 °C for 30 s. Finally, melting curve analysis was performed by cooling from 95 °C to 60 °C for 1 min (continuous signal acquisitions: 5 per °C). Relative expression of PINK1 was calculated by LightCycler software, using ACTB as internal control. Primer sequences were as follows: PINK1, 5′-TAC CGC TTC TTC CGC CAG TC-3′ (forward) and 5′-CGC CTG CTT CTC CTC GAT CA-3′ (reverse); ACTB, 5′-CAC TAT CGG CAA TGA GCG GTT C-3′ (forward) and 5′-AGC ACT GTG TTG GCA TAG AGG T-3′ (reverse).

### Immunofluorescence

Cells were incubated with 100 nmol/l MitoRed (MTR, λ _Ex/Em_ = 560/580 nm, Dojindo, Japan) at 37 °C for 30 min. The cells were then fixed with precooled 4% paraformaldehyde at room temperature for 15 min, permeabilized with 1% Triton X-100 (in PBS) at 4 °C for 6 min, blocked with 5% BSA (in PBS) at room temperature for 30 min, incubated with the anti-Parkin antibody (1:50) at 4 °C overnight, incubated with Alexa Fluor 488 (λ_Ex/Em_ = 488/545 nm)-conjugated secondary antibody (1:1000, 21206, Thermo Fisher, USA) at 37 °C for 30 min, and incubated with Hoechst 33342 (λ_Ex/Em_ = 355/465 nm) at 37 °C for 10 min. The cells were then observed with a laser scanning confocal microscope (LSCM, Zeiss, Germany) (×600).

### Glucose-stimulated insulin secretion (GSIS) test

Cells were incubated with glucose-free Krebs-Ringer buffer (KRB, Xingzhi Biotech, China) at 37 °C for 30 min. The cells were then stimulated with 25 mmol/l or 5 mmol/l glucose separately at 37 °C for 1 h. Supernatants were collected at 24 h post SDT and the concentrations of insulin in the supernatants were measured with a Rat Insulin ELISA Kit (Mercodia, Sweden) according to the manufacturer’s instructions. The OD of the samples at a wavelength of 450 nm was read using a microplate reader (BioTek, USA).

### Statistical analysis

The data are presented as the means ± standard errors of the mean (SEMs). Significance was determined using one-way ANOVA and Tukey-Kramer to determine differences between groups (level of significance *P* < 0.05).

## Results

### Effects of SDT on normal pancreatic beta cells

**Fig. 1:**
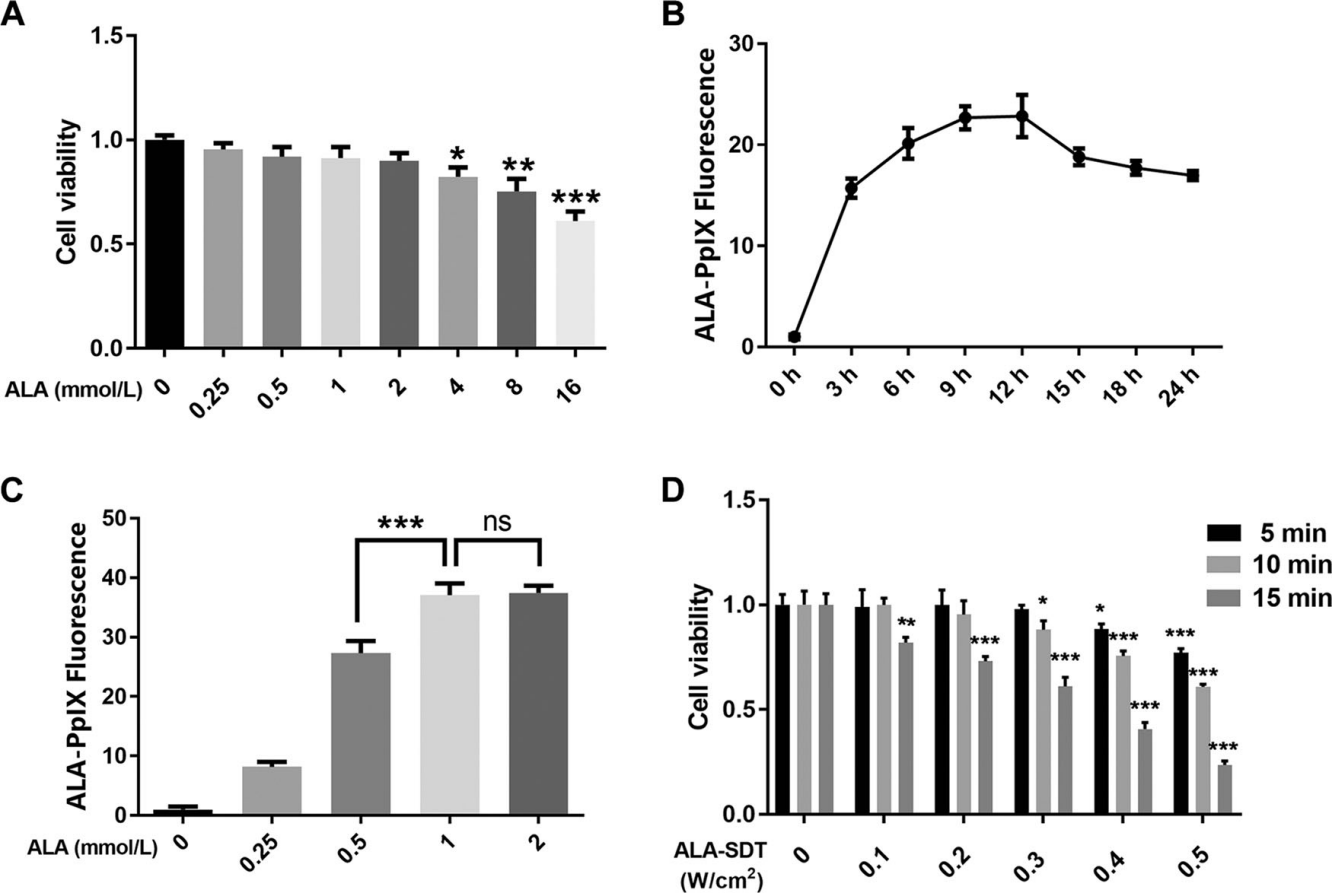
Optimal parameters of SDT to treat normal pancreatic beta cells. **a** Cell viability did not significantly change in beta cells incubated with 0.25–4 mmol/l ALA for 24 h. *n* = 6. *P < 0.05, ** *P* < 0.01, ***P < 0.001 compared to 0 mmol/l groups. **b**, **c** Accumulation of ALA-PpIX in beta cells reached peaks at the timepoint of 9 h and at the concentration of 1 mmol/l ALA. Fluorescence of ALA-PpIX was detected using fluorescence spectrophotometer and normalized to cell population. *n* = 6. ***P *<* 0.001 compared to 1 mmol/l groups. **d** Change of cell viability after 24 h post ALA-SDT is shown. Cells were preincubated with 1 mmol/l ALA for 9 h, then SDT was performed. *n* = 6. * *P* < 0.05, **P < 0.01, ***P < 0.001 compared to 0 W/cm^2^ groups. Bars = Mean ± SEM. *ALA* 5-aminolevulinic acid, *PpIX* protoporphyrin IX, *SDT* sonodynamic therapy

Whether ALA can be utilized as a sonosensitizer in beta cells was completely unknown. First, the compatibility of ALA as a sonosensitizer in beta cells was evaluated by detecting the viability of beta cells cultured for 24 h with different concentrations of ALA in culture medium. The viability of beta cells did not decrease compared with that of the control cells except for ALA concentrations greater than or equal to 4 mmol/l (Fig. 1a). Then, the accumulation of ALA in beta cells was detected by fluorescence imaging or spectrometry, as ALA can be converted to its active form, PpIX, in targeted cells, which can emit red fluorescence by light irritation. The maximal fluorescence of ALA-PpIX was respectively observed at 9 h timepoint (Fig. 1b and Fig. S1a) and at 1 mmol/l ALA concentration (Fig. 1c and Fig. S1b). To screen ALA based SDT (ALA-SDT) that does not induce cytotoxicity, beta cells were incubated with 1 mmol/l ALA for 9 h and were then treated with different ultrasound intensities (0.1–0.5 W/cm^2^). As shown in Fig. 1d and by the removal of parameters unfavorable to cells, an acoustic intensity of 0.1–0.3 W/cm^2^ and a treatment duration of 5 min were selected for the following experiments.

### SDT alleviates PA-induced beta cell injury

**Fig. 2:**
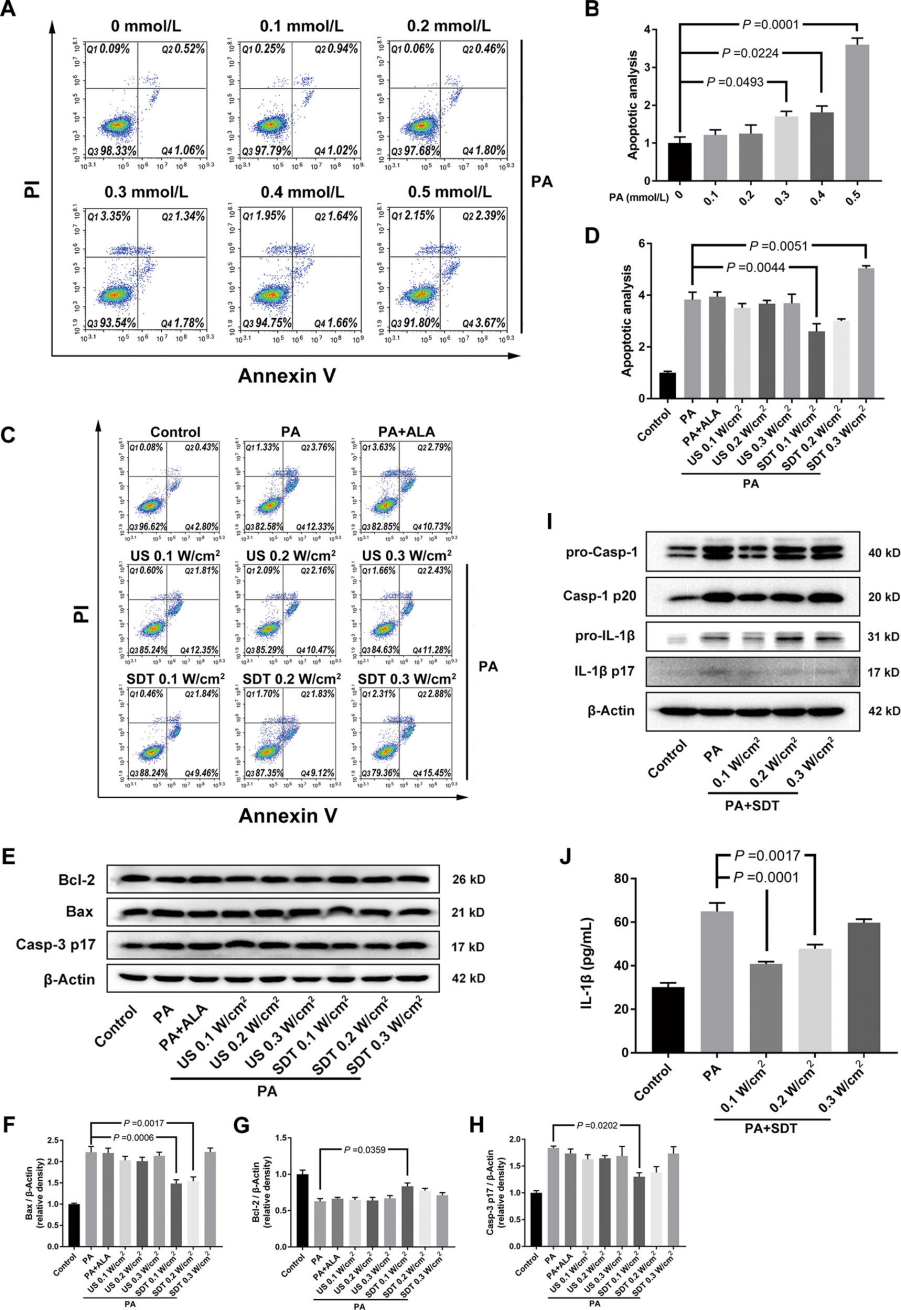
SDT alleviates PA-induced beta cell apoptosis and inflammation. **a** Model of beta cell injury was established by treating beta cells with PA for 48 h. Cell apoptosis was detected with Annexin V/PI staining by flow cytometry. Data is shown in **b** as the apoptotic ratio (Q2 + Q4) normalized to 0 mmol/l group. *n* = 3. **c**, **d** SDT inhibited apoptosis of beta cells induced by PA. Beta cells were treated with 0.3 mmol/l PA. Then, cells were treated by SDT for 5 min. Apoptosis was detected at 24 h post SDT. *n* = 3. **e**–**h** SDT downregulated proapoptotic protein levels and upregulated antiapoptotic protein levels in beta cell injury model. Cell lysates were analyzed by immunoblotting for the levels of Bcl-2, Bax, cleaved caspase-3. β-actin was used as an internal control. *n* = 4. **i**, **j** SDT attenuated proinflammatory cytokines caspase-1 and IL-1β levels. *n* = 3. Moreover, the secretion of IL-1β to supernatant was measured by ELISA. *n* = 3. Representative images are shown. Bars = Mean ± SEM. *Bax* Bcl-2 associated X Protein Bcl-2 B-cell lymphoma-2, *IL-1β* interleukin-1β, *PA* palmitic acid, *US* ultrasound

PA, the most common saturated FFA, can induce the dysfunction of beta cells both in vivo and in vitro through promoting apoptosis and inflammation via oxidative stress. A model of beta cell injury was established by treating beta cells with PA for 48 h, and apoptosis was detected by flow cytometry to ensure the successful establishment of the cell model. The results showed that PA induced significant beta cell apoptosis and that the ratio of apoptosis was dependent on the concentration of PA (Fig. 2a, b). A PA concentration of 0.3 mmol/l was applied in the following experiments to mimic the early stage of FFA overload in beta cells. SDT treatment at an acoustic intensity of 0.1 W/cm^2^ for 5 min protected beta cells by evading apoptosis (Fig. 2c, d). Moreover, the expression of proapoptotic proteins, e.g., Bax and cleaved caspase-3, were downregulated by SDT, but the expression of the antiapoptotic protein Bcl-2 was upregulated. In contrast, ultrasound alone or ALA alone did not have the above effect on beta cell injury (Fig. 2e–h). SDT treatment at 0.1 W/cm^2^ for 5 min also attenuated the levels of cleaved caspase-1 (p20) in cell lysates and mature IL-1β (p17) in both cell lysates and supernatants, but SDT at 0.2 or 0.3 W/cm^2^ did not (Fig. 2i, j).

### SDT reduces PA-induced mitochondrial dysfunction

**Fig. 3:**
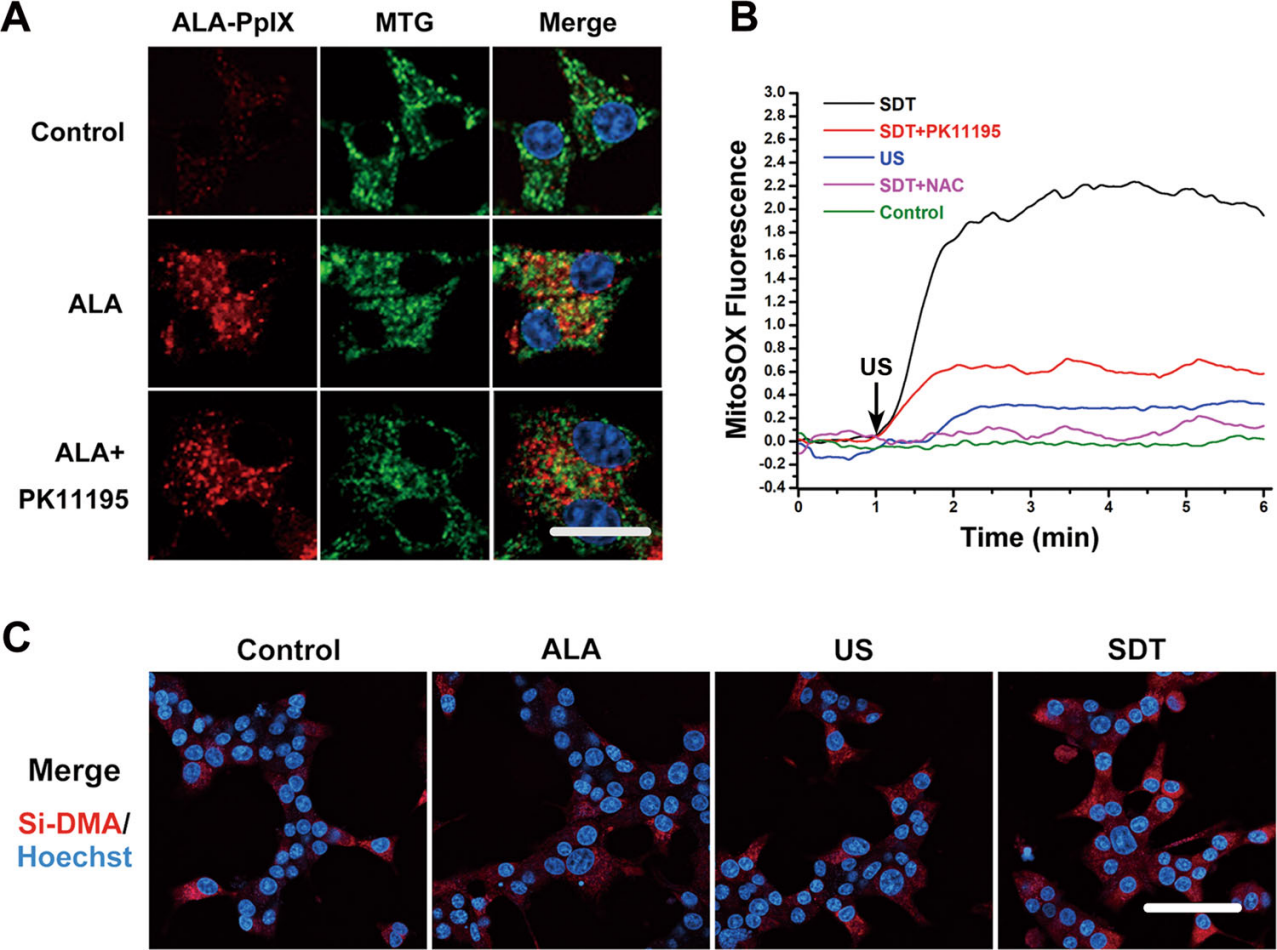
Mitochondria are potent targets of SDT in beta cells. **a** ALA-PpIX (red) localizes to mitochondria. Beta cells were preincubated with ALA. Mitochondria were stained with MTG (green) and nuclei with Hoechst 33342 (blue). *n* = 3. Scale bar is 10 μm. **b** SDT induced extensive mitochondrial ROS production. Fluorescence intensity of MitoSOX was measured with fluorescence spectrometry in real time while performing SDT. *n* = 3. MTG, Mito-Tracker Green; NAC, N-acetylcysteine; ROS, reactive oxygen species. **c** SDT induced mitochondrial singlet oxygen production. Mitochondrial singlet oxygen was stained with Si-DMA (red) and nuclei with Hoechst 33342 (blue). Cells were observed with LSCM at 0.5 h post SDT. *n* = 3. Scale bar is 30 μm

PpIX is a conserved ligand for mammalian translocator protein (TSPO), which is enriched on the outer membrane of mitochondria. Pharmacological interference with the binding of the TSPO protein via PK11195 can decrease the accumulation of PpIX in mitochondria but does not interfere with the conversion of ALA to PpIX. To validate whether TSPO is the target of PpIX in beta cells, beta cells were preincubated with PK11195 before exposure to PpIX. PpIX clearly co-localized with mitochondria in beta cells, but this phenomenon was significantly attenuated in the presence of PK11195 (Fig. 3a). ROS production might be the main mechanism of SDT and be used to evaluate the effect of SDT on beta cells. SDT induced transient large amount of mitochondrial ROS production in real time, whereas PK11195 partly blocked the ROS production induced by SDT (Fig. 3b). Singlet oxygen might play a significant role in bioeffects elicited by SDT. As shown in Fig. 3c, SDT obviously induced mitochondrial singlet oxygen production compared to ALA groups or US groups at 0.5 h post SDT.

**Fig. 4:**
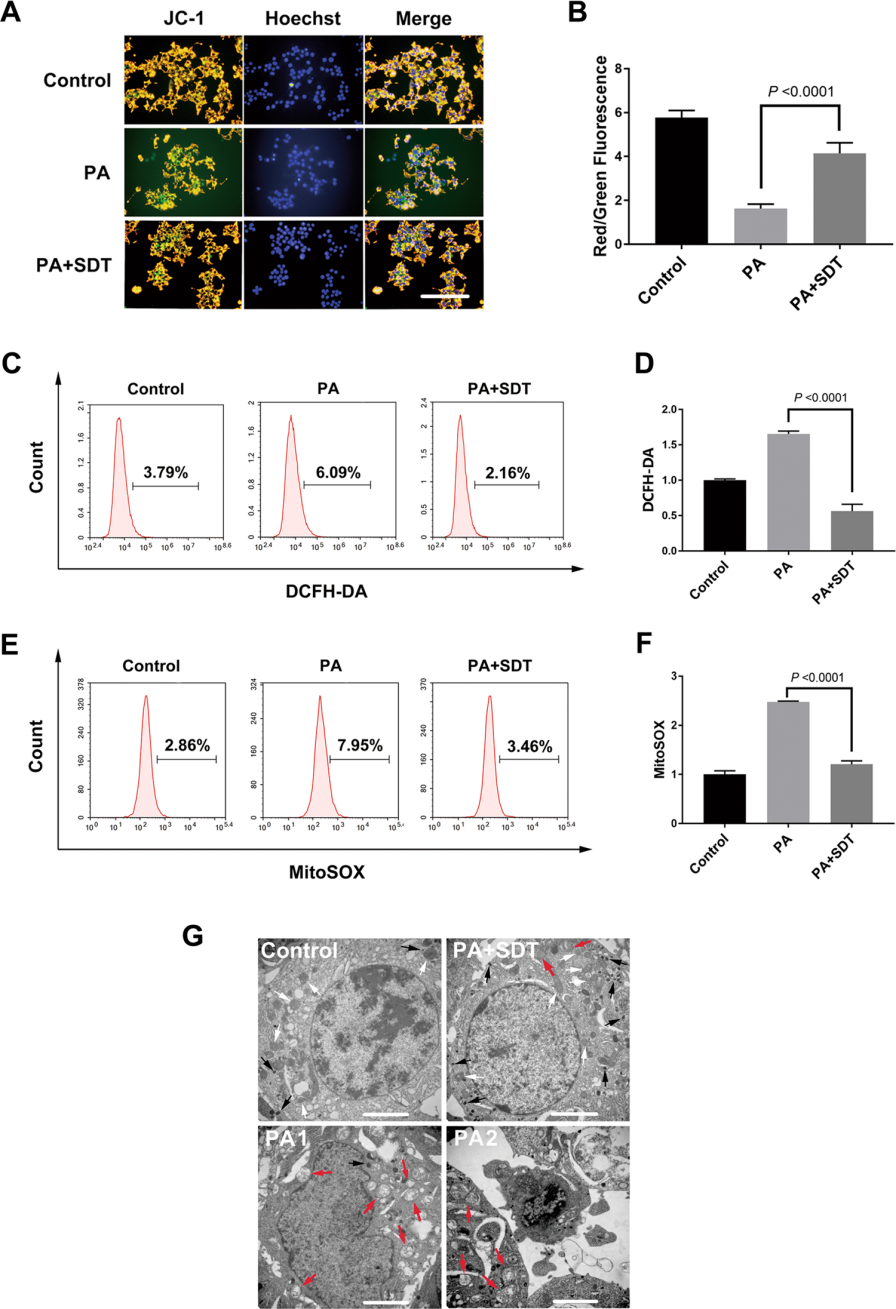
SDT reduces PA-induced mitochondrial dysfunction. **a** SDT recovered the loss of Δψm induced by PA. After 24 h post SDT, cells were stained with JC-1. Red-fluorescence indicates normal Δψm, green indicates loss of Δψm and blue indicates nuclei. Scale bar = 100 μm. Date are shown in **b** as the ratio of red-fluorescent cell number to green-fluorescent cell number. *n* = 4. **c**–**f** SDT inhibited both intracellular and mitochondrial ROS production. ROS levels were analyzed by flow cytometry at 24 h post SDT. *n* = 3. **g** SDT improved mitochondrial morphology. Images were observed with TEM at 24 h post SDT. The white arrows indicate normal mitochondria, the red arrows indicate swollen mitochondria and the black arrows indicate secretory granules. *n* = 3. Scale bar = 2 μm. Representative images are shown. Bars = Mean ± SEM. *TEM* transmission electron microscope, *Δψm* mitochondrial membrane potential

As shown above, SDT might target the mitochondria in beta cells. However, whether SDT can modulate the quality of mitochondria in beta cells needed to be further evaluated. Cells were collected and detected at 24 h post SDT. First, the change in the Δψm, detected by JC-1 staining, is presented as the ratio of red-fluorescent cell number to green-fluorescent cell number. As shown in Fig. 4a, b, the Δψm in beta cells was significantly reduced by PA, but interestingly, the loss of the Δψm could be recovered by SDT treatment. Moreover, increased production of mitochondrial ROS and total intracellular ROS was observed in the PA group, and the production of both was inhibited by SDT (Fig. 4c–f, Fig. S2a, b). Finally, the ultrastructure of PA-stimulated beta cells with or without SDT treatment was observed with TEM. Many swollen mitochondria and apoptotic bodies were observed in PA-injured beta cells, which suggested that the beta cells underwent apoptosis. In contrast, mitochondrial morphology improved, and the quantity of mitochondria and secretory granules, which are important components of beta cells, increased in PA-injured beta cells treated with SDT (Fig. 4g).

### SDT induces PINK1/Parkin-dependent mitophagy

**Fig. 5:**
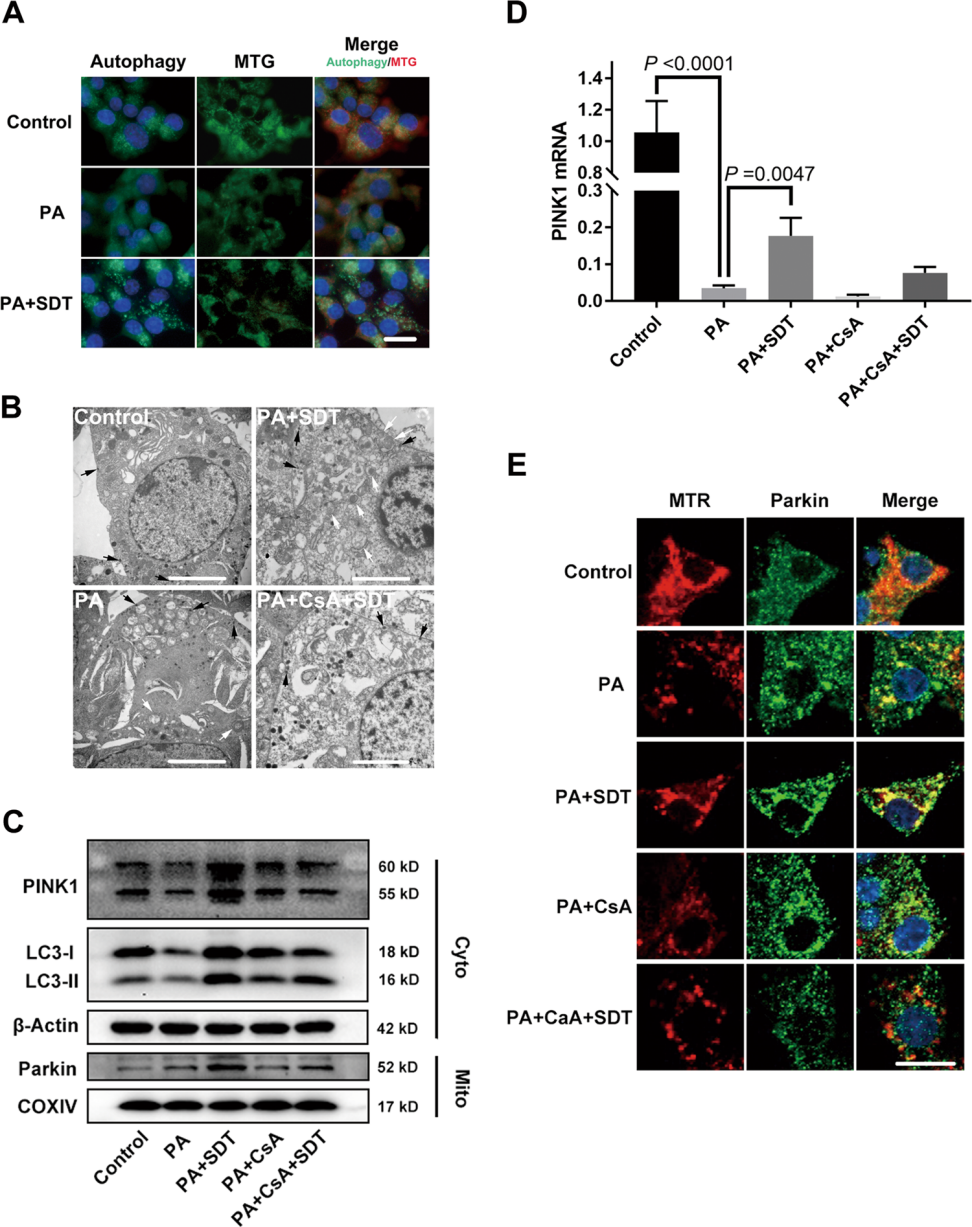
SDT induces PINK1/Parkin-dependent mitophagy and rescues the expression of PINK1. **a** SDT increased the colocalization of autophagosomes and mitochondria. Autophagosomes were labeled with an autophagy assay kit (blue-green) and mitochondria with MTG (green and changed to red after merge) and nuclei with Hoechst (blue). Scale bar = 10 μm. **b** SDT increased the colocalization of mitochondria and bilayer vesicles at 0.5 h post SDT. Mitochondria encapsulated by bilayer vesicles are indicated by white arrows, and black arrows indicate secretory granules. Scale bar = 2 μm. **c** SDT upregulated mitophagy related protein levels. After 0.5 h post SDT, cell lysates and mitochondrial extracts were analyzed by immunoblotting for the levels of PINK1, LC3B, Parkin. β-Actin and COXIV were used as internal controls. *n* = 4. **d** SDT restored the expression level of PINK1 mRNA inhibited by PA. Bars = Mean ± SEM, *n* = 3. **e** SDT induced the translocation of Parkin from the cytoplasm to mitochondria. Parkin is shown by green and mitochondria by red. Scale bar = 10 μm. Representative images are shown. *COXIV* Cytochrome c oxidase IV, *CsA* cyclosporin A, *LC-3* microtubule-associated protein 1 light chain 3, *MTR* MitoRed, *PINK1* PTEN-induced putative kinase 1

Mitophagy selectively removes damaged mitochondria and thus participates in mitochondrial quality control. However, the malfunction of mitophagy may cause the destruction of beta cells and eventually lead to the development of diabetes. In this study, we found puncta (autophagosomes) were significantly reduced by PA treatment, and SDT treatment increased autophagosomes and colocalization with mitochondria (Fig. 5a); moreover, the encapsulation of mitochondria by bilayer vesicles was significantly increased by SDT but was inhibited by adding the mitophagy inhibitor CsA to the medium (Fig. 5b). Next, we evaluated the expression of mitophagy related protein. SDT treatment upregulated the expression of cytoplasmatic PINK1 and LC3-II, which were downregulated by PA, and that of Parkin in mitochondria. However, the upregulation of mitophagy related protein by SDT was inhibited by CsA (Fig. 5c).

To determine whether PINK1 mediated the induction of mitophagy by SDT, the expression of PINK1 mRNA was next quantified with RT-qPCR. Compared to untreated control cells, PA-treated beta cells exhibited downregulated expression of PINK1 mRNA, and SDT restored the expression level of PINK1 mRNA. However, the upregulation of PINK1 mRNA by SDT was blocked by the mitophagy inhibitor CsA (Fig. 5d). Since PINK1 and Parkin can selectively remove damaged mitochondria, and this process is initiated by the anchoring of PINK1 to the outer membrane of mitochondria with a low Δψm, we explored the recruitment of Parkin in mitochondria. SDT promoted the colocalization of the Parkin protein with mitochondria; however, the SDT-induced translocation of Parkin from the cytoplasm to mitochondria was blocked by CsA (Fig. 5e). The above results suggested that SDT induced mitophagy by rescuing the PINK1 deficiency at the level of transcriptional regulation in damaged beta cells.

### SDT protects beta cells by suppressing inflammation through mitophagy

**Fig. 6:**
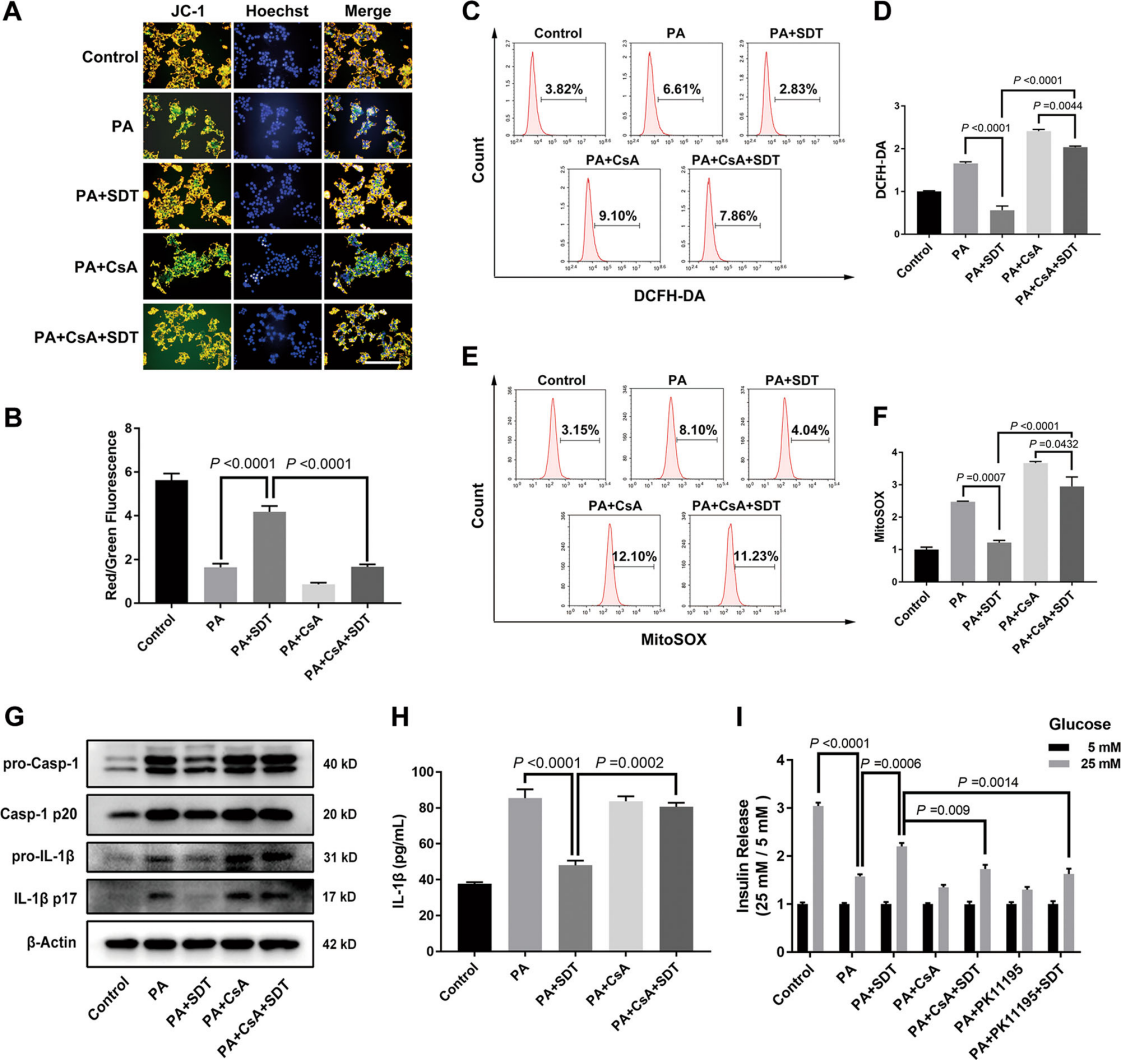
SDT protects beta cells by suppressing mitochondrial dysfunction and inflammation through mitophagy. **a** The recovery of Δψm by SDT was attenuated by CsA. Scale bar = 100 μm. Date are shown in **b** as the ratio of red-fluorescent cell number to green-fluorescent cell number. *n* = 4. **c**–**f** The inhibition of mitochondrial ROS production by SDT was abolished by CsA. ROS was detected at 24 h post SDT. *n* = 3. **g**, **h** The downregulation of proinflammatory cytokine levels and IL-1β secretion was abrogated by CsA. *n* = 3. **i** SDT ameliorated the defective insulin secretion from PA injured beta cells. However, this favorable effect was blocked by PK11195 or CsA. Insulin secretion was evaluated by GSIS test. *n* = 3. Representative images are shown. Bars = Mean ± SEM

The effect of SDT on mitochondria and the function of beta cells treated with or without CsA was further evaluated at 24 h post SDT. According to JC-1 staining results, the Δψm improvement induced by SDT was attenuated by CsA (Fig. 6a, b). Mitochondrial and cytosolic ROS were detected by flow cytometry and fluorescence imaging, and the effect of SDT on the decrease of ROS in both mitochondria and the cytoplasm was reduced by the addition of CsA (Fig. 6c–f, Fig. S2a, b). These results suggested that mitophagy played an important role in the protection of mitochondria in beta cells. As SDT improved the quality of mitochondria and reduced ROS from malfunctional mitochondria, SDT might also reduce inflammation in beta cells. The levels of the inflammatory factors e.g., caspase-1 and IL-1β in cell lysates and the level of IL-1β in supernatants were significantly downregulated by SDT, and this effect was abrogated by CsA (Fig. 6g, h). A glucose-stimulated insulin secretion experiment was performed to evaluate the change in the function of damaged beta cells treated with SDT. The defective secretion of insulin from beta cells was significantly ameliorated by SDT treatment. However, both blocking the effect of SDT on mitochondria by PK11195 treatment and blocking mitophagy by CsA treatment inhibited the favorable effect of SDT on insulin secretion. Thus, the above results demonstrated that SDT protected mitochondria in damaged beta cells by inducing mitophagy, also reducing further cell injury and improving insulin secretion.

## Discussion

**Fig. 7:**
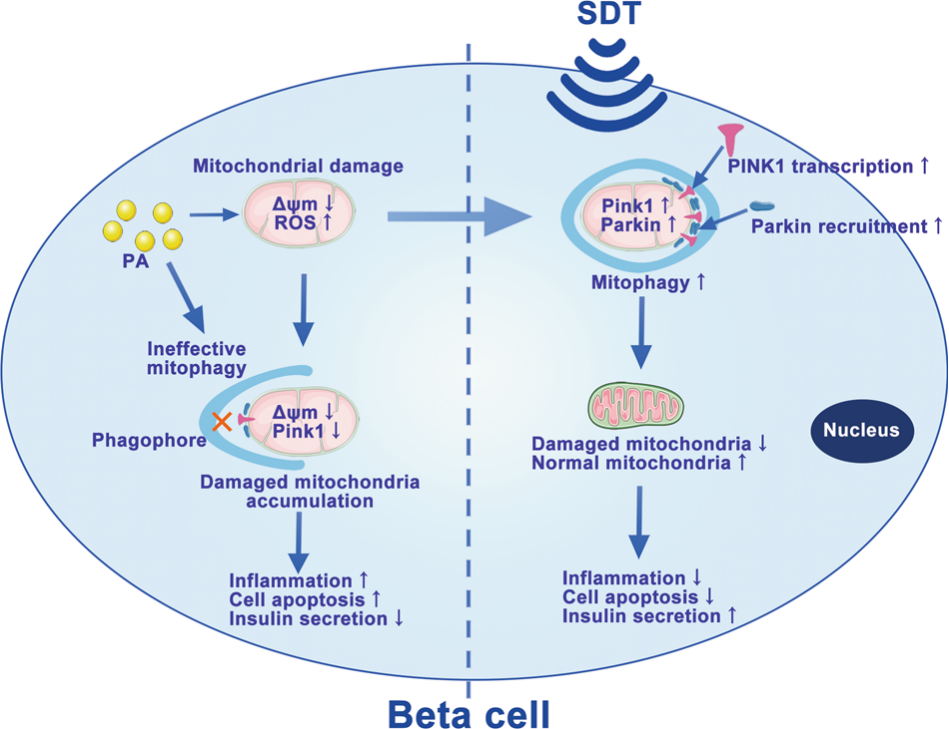
Schematic of SDT inhibits PA-induced beta cell injury via PINK1/Parkin-dependent mitophagy. In beta cells, PA damages mitochondria and mitophagy. Damaged mitochondria accumulate in cells due to inadequate mitophagy and produce massive ROS, thus promote inflammation and cell apoptosis. SDT restores the PINK1/Parkin-dependent mitophagy through upregulation of PINK1 expression and recruitment of Parkin to mitochondria. Thus, SDT suppresses inflammation and cell apoptosis and promotes insulin secretion. *PA* palmitic acid, *PINK1* PTEN-induced putative kinase 1, *ROS* reactive oxygen species, *SDT* sonodynamic therapy

An overabundance of glucose and FFAs in pancreatic beta cells leads to the production of ROS, which is the underlying cause of the development of islet beta cell dysfunction, insulin deficiency and T2DM^32^. Conventional treatments, including oral medications such as metformin or insulin injections, have transient effects but not long-term effects on the improvement of beta cell function^9,11,12^. Therefore, continuing to develop new treatment programs that can persistently improve the function of beta cells is necessary. Rat insulinoma RIN-m5F cells are a widely used model of pancreatic islet cells with glucose-stimulated insulin secretion and can be used to evaluate fatty acid-induced lipotoxicity^33^. This study applied SDT, a nontoxic and noninvasive targeted treatment, to the treatment of beta cells damaged by PA treatment. SDT potently inhibited the toxic effects of PA, including mitochondrial damage, cell injury, and the impairment of insulin secretion from beta cells. The mechanism of SDT likely operated by rescuing the PINK1/Parkin-dependent mitophagy blocked by PA, as SDT upregulated the expression of PINK1 mRNA, which was transcriptionally inhibited by PA (Fig. 7). These results suggested that SDT had great potential to treat T2DM by protecting islet beta cells via mitophagy.

As human beings age, the regeneration capacity of islet beta cells is significantly reduced, and both the number and function of beta cells gradually deteriorate^34,35^. In particular, high concentrations of glucose and FFAs accelerate the loss and malfunction of beta cells, eventually leading to the failure of insulin secretion^14,15^. Thus, the progression of diabetes depends on the degree of beta cell failure. Although some drugs such as sulfonylurea, glucagon-like peptide-1 receptor agonists and dipeptidyl peptidase-4 inhibitors can control blood sugar by stimulating endogenous insulin secretion, their extended use may also be harmful to injured beta cells by increasing the metabolic burden and accelerating the functional depletion of beta cells^11^. We were encouraged by the finding that SDT inhibited PA-induced apoptosis of beta cells and improved insulin secretion from PA-treated beta cells. The effects of SDT on insulin secretion might result from the promotion of cell proliferation or the upregulation of insulin secretion.

During cell aging or stimulation by toxic substances, excess ROS can damage cellular components, such as DNA, RNA, carbohydrates, proteins and lipids, in specific signaling pathways with organelle-specific functions^36,37^. In addition to aging, the high sugar and high fat levels in T2DM may cause the accumulation of islet amyloid polypeptide (IAPP), forming toxic substances in beta cells and then inducing ER and oxidative stress, inflammation and islet beta cell failure^36^. Albeit without exploring the whole process of oxidative stress, we investigated the direct indicators of oxidative stress, e.g., intracellular ROS and inflammatory factors. This study showed that mitochondria in beta cells damaged by PA were swollen and deformed, the mitochondrial crests disappeared, and the Δψm decreased significantly. Furthermore, the level of intracellular ROS, especially mitochondrial ROS, increased significantly, and caspase-1 and IL-1β were activated. These results were consistent with those of previous reports showing that lipid toxicity can activate the NOD-like receptor 3 (NLRP3) inflammasome through mitochondrial damage to interfere with insulin secretion and signal transduction^38,39^. As ALA accumulated in the mitochondria of beta cells, ALA-SDT might also modulate the function of mitochondria. Indeed, the toxic effects of PA on mitochondria, such as mitochondrial swelling, the decrease in the Δψm and the production of mitochondrial ROS, as well as the expression of inflammatory factors, were significantly inhibited by SDT. Moreover, the quantity of mitochondria and intracellular secretory granules increased significantly after SDT. These results suggested that SDT could be a valid treatment method for beta cells and mitochondria are important subcellular targets of SDT in beta cells. The improved mitochondria might provide enough energy for the activity of insulin signaling pathways, thus promoting the recovery of insulin secretion^39^. However, the underlying mechanism of mitochondrial improvement by SDT is unclear.

Both mitochondrial damage and repair processes coexist in mitochondria challenged by oxidative stress^40,41^. Oxidative stress is usually followed by the activation of autophagy, which might be a protective mechanism^42–44^. Mitophagy is one of the most important pathways that can control the quality of mitochondria by selectively removing damaged mitochondria^45,46^. However, defective mitophagy, which might be caused by long-term oxidative stress, is a common feature of several diseases, such as diabetes, atherosclerosis and neurodegenerative disease^46–48^. Defective mitophagy occurs in the beta cells of obese T2DM patients, and PA treatment can lead to the dysfunction of mitophagy in beta cells in vitro^47^. In addition, defective mitophagy contributes to the production of mitochondrial ROS, the activation of NLRP3-dependent proinflammatory responses and the exacerbation of lipotoxicity^49^. However, the underlying mechanism of PA-induced defective mitophagy has not been fully clarified.

In this study, we found that PA inhibited the transcription of PINK1, thus blocking the recognition of damaged mitochondria by autophagosomes. The PINK1/Parkin pathway is one of the important signaling pathways initiating the mitophagy of mitochondria with a low Δψm^47^. Full-length PINK1 (60 kDa) can be inserted into mitochondria but with different outcomes. When inserted into a normal mitochondrion, full-length PINK1 is cleaved into its short form (55 kDa) without the N-terminus and is then degraded in the cytoplasm. Alternatively, PINK1 can be incorporated into a mitochondrion when the Δψm decreases; then, PINK1 recruits Parkin to the outer membrane of mitochondria and initiates mitophagy^50^. In this study, SDT restored the expression of PINK1 mRNA, which was blocked by PA, and promoted the removal of damaged mitochondria by mitophagy. Moreover, the mitophagy inhibitor CsA attenuated the protective role of SDT in beta cells pretreated with PA.

ROS refers to components containing oxygen that are produced by oxidative metabolism, such as superoxide anion (O_2_^•^^−^), hydrogen peroxide (H_2_O_2_), and singlet oxygen. The baseline level of ROS plays an important role in cell signaling and is involved in normal cell metabolism and homeostasis^51,52^. However, elevated intracellular ROS can induce oxidative damage to lipids, proteins and nucleic acids and can lead to cell dysfunction and even cell death^53,54^. Therefore, the amount of ROS production and the relative defect in antioxidative capacity determine the major role of ROS in specific cells^55^.

SDT induced transient large amount of mitochondrial ROS production in real time, and also induced obvious production of mitochondrial singlet oxygen at 0.5 h post treatment, but not in ALA or US groups. The production of singlet oxygen may be caused by the excitation of PpIX co-localized on mitochondria by sonoluminescence^56^. Whether sonoluminescence occurs and the mechanism of sonoluminescence is worthy of further study. The results suggested that SDT targeted the mitochondria of beta cells and played biological roles by inducing transient ROS production, consistent with our previous findings in macrophages^57,58^. However, a reduction of both intracellular and mitochondrial ROS was observed at 24 h post SDT. Under this circumstance, the reduction of ROS suggested that SDT probably improved the function of mitochondria or cells.

We hypothesized that ROS plays different roles in different circumstances. Long-term oxidative stress might damage beta cells by causing inflammation, apoptosis or the loss of self-protection, while transient exposure to a large amount of ROS might reactivate the protective signaling pathway(s). For example, PINK1 transcription might be reactivated in PA-damaged beta cells by ROS produced by SDT treatment, which thus played an important protective role via mitophagy. However, the mechanism by which SDT-produced ROS acts as a signal to restore PINK1 transcription in order to activate mitophagy requires further research and evidence.

In summary, we have shown for the first time that SDT protects beta cells against PA damage by reducing the ratio of apoptosis and improving insulin secretion in vitro. SDT likely inhibits the accumulation of damaged mitochondria and inflammatory response by inducing transient large amount of mitochondrial ROS and PINK1/Parkin-dependent mitophagy. SDT relieves the transcriptional inhibition of PINK1 by PA and thus increases PINK1 transcription and activates mitophagy (Fig. 7). Thus, our study provides theoretical support for this potential treatment protocol and for further studies in vivo.

## Supplementary information

Supplemental Materials
