# Supplementary material for: Sonodynamic therapy inhibits palmitate-induced beta cell dysfunction via PINK1/Parkin-dependent mitophagy
Source: Cell Death Dis. 2019 Jun 11;10(6):457. doi: 10.1038/s41419-019-1695-x (PMC6560035; doi:10.1038/s41419-019-1695-x)
Supplement: Supplementary file 1 — Supplemental Materials [file 41419_2019_1695_MOESM1_ESM.docx]

**Supplemental Materials**

**Table S1 The maximum pressure amplitude (0-peak pressure) (*p*_MAX_) values in culture media at different ultrasound intensity.**

| **Intensity (W/cm^2^)** | ***p*_MAX_ (kPa)** |
| --- | --- |
| 0.1 | 60.593 |
| 0.2 | 89.967 |
| 0.3 | 136.729 |
| 0.4 | 241.745 |
| 0.5 | 371.674 |


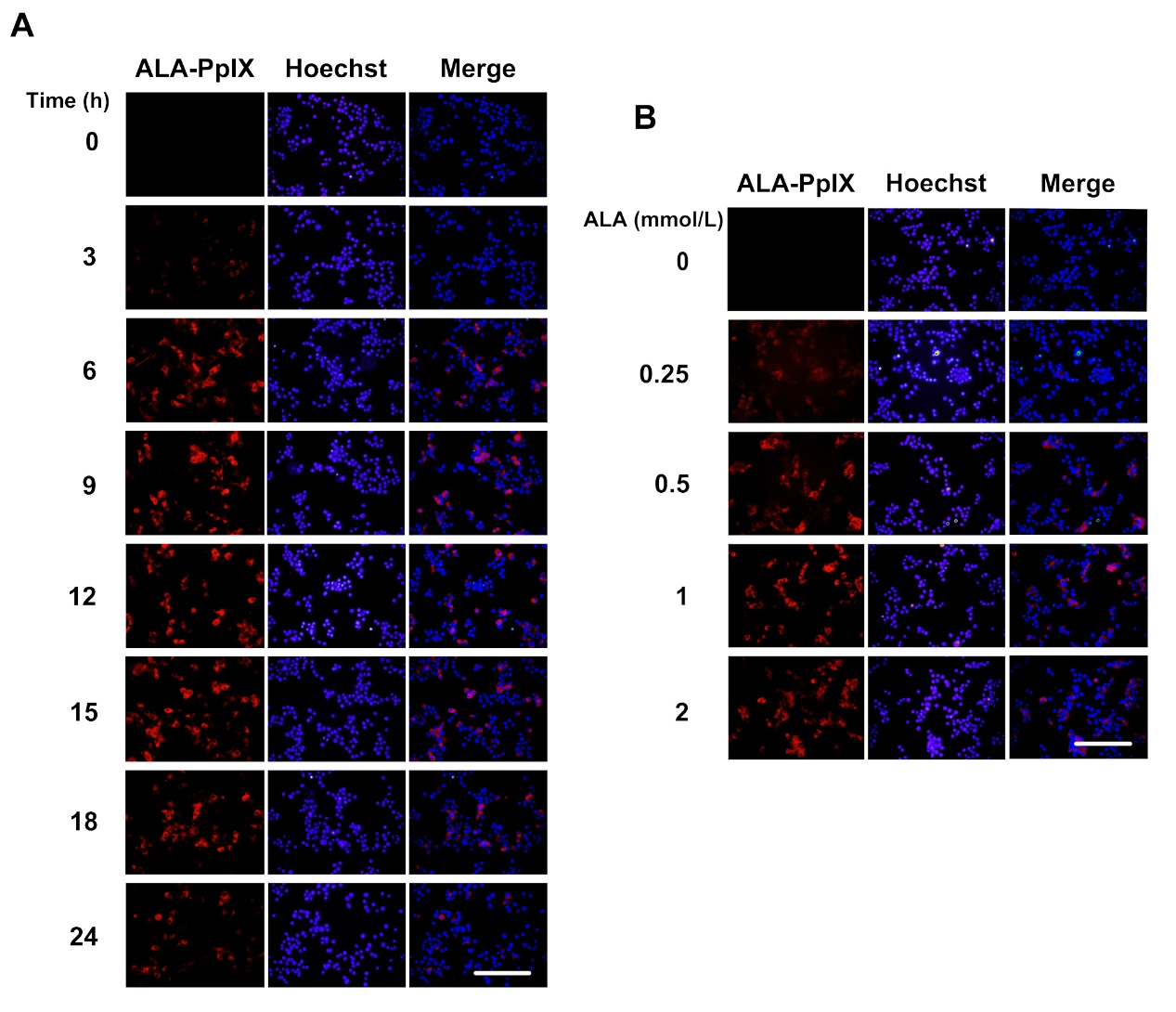


**Fig. S1 Accumulation of ALA-PpIX in** **beta cells is dependent on time course or ALA concentration.** **a** Time-dependent PpIX fluorescence**. b** Concentration-dependent PpIX fluorescence. Representative images are shown. ALA-PpIX is shown in red and Hoechst in blue. Scale bar = 100 μm.


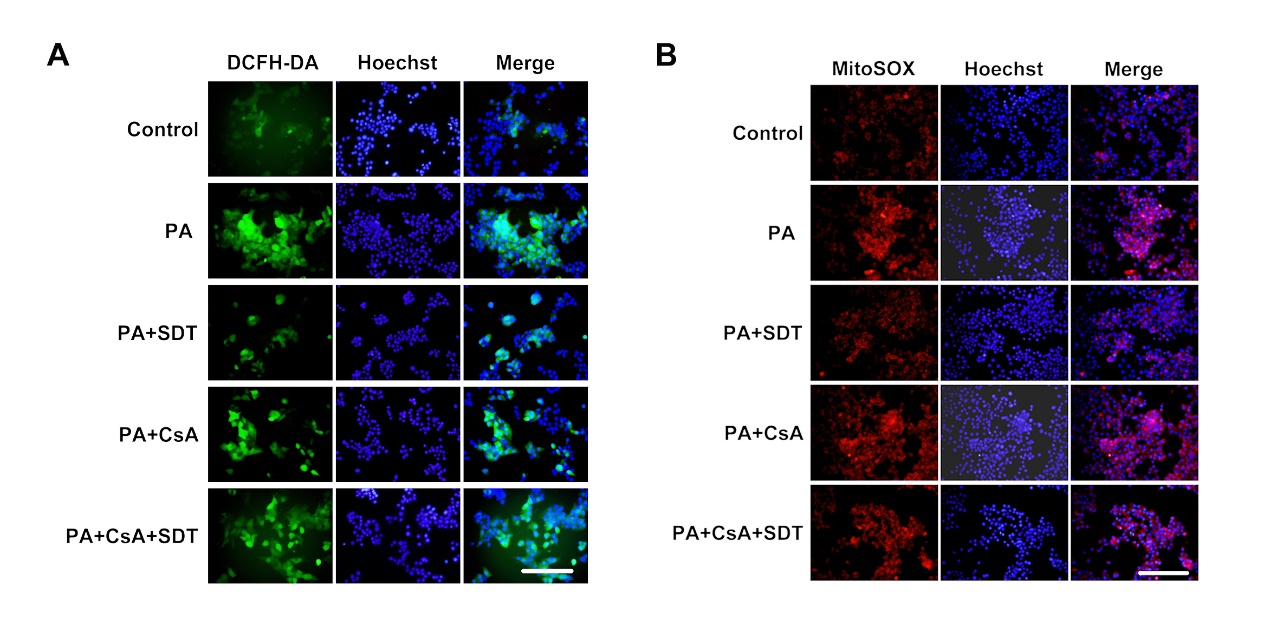
**Fig. S2 SDT reduces ROS production through mitophagy.** Intracellular ROS was stained with DCFH-DA **a** and mitochondrial ROS with MitoSOX **b**. Representative images are shown. DCFH-DA is shown in green, MitoSOX in red and Hoechst (nuclei) in blue. Scale bar = 100 μm.
